# Systemic immune inflammatory index and mortality in chronic kidney disease

**DOI:** 10.3389/fendo.2025.1605543

**Published:** 2025-09-03

**Authors:** Yanshuang Ma, Yang Yu, Zhongdan Jia, Hushan Wang, Mingli Sun

**Affiliations:** ^1^ Department of Hyperbaric Oxygen, First Hospital of Jilin University, Changchun, Jilin, China; ^2^ Department of Anesthesiology, First Hospital of Jilin University, Changchun, Jilin, China; ^3^ Department of Radiation Oncology, First Hospital of Jilin University, Changchun, Jilin, China

**Keywords:** chronic kidney disease, systemic immune-inflammatory index, cardiovascular disease, cancer mortality, all causes mortality

## Abstract

**Background:**

Chronic kidney disease (CKD) is common and linked to higher mortality rates, but its relationship with the systemic immunoinflammatory index (SII) remains unclear, highlighting the need for further research.

**Methods:**

The SII is calculated by multiplying the counts of platelets and neutrophils, followed by dividing that product by the lymphocyte count. A diagnosis of CKD is made when the estimated glomerular filtration rate (eGFR) is below 60 mL/min/1.73 m². To further analyze the data, a multivariable Cox regression analysis, along with subgroup assessments, was performed. Analysis of survival data and threshold effects suggests that SII is a crucial independent factor related to mortality from all causes and cardiovascular issues in individuals with chronic kidney disease.

**Results:**

The study comprised a total of 46,620 individuals, with a weighted average age (standard error) of 47.00 (0.18) years. Among individuals over 20 years old suffering from CKD, the log-transformed SII demonstrated a nonlinear relationship, revealing a U-shaped correlation with the mortality rates associated with all causes as well as cardiovascular diseases (CVD). When SII.log as the log values rise, the likelihood of dying from all causes and cardiovascular issues initially shows a decline. Nevertheless, this pattern shifts, leading to an increased risk of mortality once a certain limit is surpassed. The evaluation of threshold impacts identified important levels starting at 6.06 and 6.25, which corresponded to the lowest observed mortality risk when evaluated through the SII.log values. The likelihood of mortality from all causes escalated once these limits were surpassed (HR 0.75, 95% CI 0.64-0.88; HR 1.74, 95% CI 1.55-1.95). The likelihood of mortality due to CVD also elevated (HR 1.01, 95% CI 0.78-1.31; HR 1.91, 95% CI 1.50-2.44). Higher SII levels correlated with decreased survival and longevity. In CKD patients over 45 years, SII reliably predicted all-cause mortality (statistically significant) and was linked to cardiovascular and cancer deaths (not statistically significant).

**Conclusions:**

SII is an easily obtainable marker that may predict mortality in CKD patients over 45. More longitudinal research is needed to confirm its link with mortality rates (all causes, cardiovascular, and cancer) due to current limitations.

## 1 Introduction

Due to the global population growth and aging, the prevalence and incidence of diabetic kidney disease (DKD) and CKD have been increasing year by year over the past few decades (1–3). Over the past 20 years, the number of deaths related to chronic kidney disease (CKD) has increased significantly (4). It is notable that the global ranking of CKD has has risen to the 9th position globally (5). This reflects the increasingly serious impact it has on global mortality as a major contributing factor (4). The presence of chronic kidney disease (CKD) is marked by a reduction in glomerular filtration rate or long-term proteinuria that persists beyond a duration of three months. One of the key underlying conditions is diabetes mellitus and hypertension, along with issues related to glomerular inflammation, which significantly contribute to the development of chronic kidney disease (CKD). Addressing proteinuria is vital for improving outcomes among patients with CKD (6).This condition poses a significant public health issue globally, marked by a gradual deterioration of renal function (7). In some countries, such as China, the prevalence of CKD is also showing a significant upward trend. According to the Global Burden of Disease Study in 2019, the prevalence of CKD in China increased from 6.7% in 1990 to 10.6% in 2019, and is projected to further rise to 11.7% by 2029 (3). This upward trend is closely related to the increase in related chronic diseases such as obesity and diabetes in the Chinese population (8).This series of investigations highlights the critical importance of accurately identifying persons who are susceptible to chronic kidney disease (CKD).While conventional risk factors associated with cardiovascular diseases (CVD) and cancer are frequently observed among CKD patients, they fail to fully account for the increased mortality rates within this population. Models developed based on traditional clinical parameters are ineffective in forecasting long-term prognoses, revealing a critical need for effective risk stratification tools (9, 10).

Increased mortality in patients with chronic kidney disease (CKD) may also be related to inflammation. Plasma levels of multiple inflammatory biomarkers such as IL-1β, IL-6, TNF-α, and hs-CRP were higher in participants with lower eGFR and higher UACR and higher inflammation scores. Inflammatory markers were negatively correlated with renal function indexes and positively correlated with albuminuria (11). It can be seen that inflammatory markers are closely related to the prognosis of CKD. There is growing interest in inflammatory markers. In 2014, a comprehensive new inflammatory biomarker was reported. The SII is recognized as an index that can be derived from blood samples collected through less invasive procedures, which can indicate not only the local immune dynamics within the body but also reflect the overarching inflammatory profile (12–15). This indicator was originally used to assess the prognosis of patients with liver cancer (16). It has been recognized recently as a significant inflammatory indicator for a range of cardiovascular conditions (CVD) (17–20). SII can serve as a biomarker for disease activity, and it is also used to assess and predict the incidence of systemic lupus erythematosus (SLE) (21). More importantly, recent studies have shown that systemic inflammatory immune indicators, as this new type of inflammatory marker, may be closely related to chronic kidney disease (22). In a study related to CKD patients, it was found that an increase in SII levels at the time of admission had a significant impact on overall mortality and specific mortality (23). However, most existing SII studies have focused on hospitalized patients. A large, multi-center, longitudinal, real-world study showed that the SII level was closely related to the risks of all-cause mortality, cardiovascular mortality and tumor mortality in hospitalized CKD patients. Incorporating the SII into the traditional risk factor model could improve the clinical prediction of long-term prognosis (23).However, it is also important to explore indicators related to the prognosis of outpatients with CKD. One study has already pointed out that outpatient interventions offer the possibility of alternatives to emergency and inpatient care for managing acute complications of chronic diseases. However, interventions are not primarily targeted at patients with CKD (24).

The above studies indicate that there is an urgent need for a monitoring tool that can be used to monitor and evaluate acute complications in outpatients with CKD. As a simple, practical, easily accessible and economical biomarker, SII has significant advantages in predicting the prognosis of outpatients with CKD. Firstly, compared with the individual counts of neutrophils, platelets or lymphocytes, SII is relatively stable because it is calculated from these three. Secondly, SII integrates inflammatory response and immune regulation, making it more comprehensive. Finally, SII is an economical, practical, low-cost and easily accessible parameter (25). However, the role of SII in the early warning of CKD in outpatients has not been fully clarified. So far, further research is needed to clarify the relationship between CKD-related mortality and the systemic inflammation index. The relationship between SII and total mortality, CVD and cancer mortality in patients with chronic kidney disease was explored using the NHANES public database.

## 2 Material and method

### 2.1 Data sources

NHANES provides a national representation of the health and nutritional status of non-institutionalized U.S.civilians. For a comprehensive understanding, it is advisable to consult the NHANES methodology guide. Information regarding demographics, dietary habits, and health-related inquiries was gathered through interviews conducted in households as part of the NHANES. Samples of blood were collected at mobile health screening sites. The National Center for Health Statistics Research Ethics Review Board approved the NHANES. A consent form, duly signed by each participant, was acquired. This model was exempt from review because it utilized an open de-identified dataset that did not contain personal identification information.

### 2.2 Study participants

This study is based on the Nhanes database. Therefore, the data of the population involved in this study is based on the data registered in the database registry. This research drew from data spanning ten cycles of the NHANES conducted between 1999 and 2018. The study included participants aged 20 years and older (n = 55,081), excluding pregnant individuals (n = 1,303) and those lacking complete data on urinary albumin-to-creatinine ratio (UACR), systemic immune-inflammation index (SII), and estimated glomerular filtration rate (eGFR). In total, the study encompassed 46,620 individuals (**Supplementary Figure S1**).

### 2.3 Definition of CKD

CKD is categorized according to etiology, eGFR (G1-G5), and proteinuria (A1-A3), which is called CGA staging (26, 27). However, in the survey, most individuals were measured only once, and when proteinuria was measured again, the time of two urine sample collections could be different. In order to reduce bias, a single measurement of UACR was employed in place of continuous 24-hour proteinuria monitoring, reflecting established methodologies in the field (28–30). The eGFR index is typically evaluated using the CKD Epidemiology Collaboration formula, a widely accepted measure of renal function, though it is not a novel assessment method. (31). UACR was categorized into three groups: A1 (<30 mg/g or <3 mg/mmol), A2 (30–300 mg/g or 3–30 mg/mmol), and A3 (>300 mg/g or >30 mg/mmol). In addition, eGFR was categorized into G1 normal or above normal (≥90 mL/min/1.73 m^2^), G2(60-89), G3a (45–59), G3b (30–44), G4 (15–29), and G5 renal failure (<15). CKD was defined as UACR of A2 level and eGFR of G3a level or worse.

### 2.4 The meaning of SII

SII = P * N/L cells/µL, where P, N, and L are peripheral blood platelet, neutrophil, and lymphocyte counts, respectively. Peripheral blood cell counts were monitored by medical professionals and measured on an instrumental analyzer (16).

### 2.5 Evaluation of results

The primary results involved fatalities from all causes, cardiovascular diseases, and cancers among the participants who were enrolled. Individuals involved in the NHANES study from 1999 to 2018 were tracked until the end of the year 2019, starting from their date of entry into the study. Data regarding mortality were sourced from death certificate records of the National Death Index by matching information such as the social security number, individual name, gender, race/ethnicity, date of birth, birth status, and residency status (32).

### 2.6 Covariates

We selected and adjusted the covariates based on previous studies. The variables taken into consideration comprised age, gender (male or female), racial/ethnic categories (Mexican American, Other Hispanic American, Non-Hispanic White, among others), relationship status (married/cohabiting, widowed/divorced/separated/unmarried), educational attainment (less than high school, high school or higher), drinking habits (yes or no), body mass index (BMI), the ratio of household income to poverty level (PIR), along with the existence of conditions such as hypertension, diabetes, heart disease, and cancer, with each being categorized as either present or absent (33, 34).PIR was detailed and graded as <1.3, 1.3-3.5, and >3.5. Cancer referred to any form of malignancy that was diagnosed through clinical evaluation. A history of hypertension was recognized when an individual had a systolic blood pressure (SBP) at or above 140 mmHg and/or a diastolic blood pressure (DBP) of 90 mmHg, or had received a medical diagnosis from a healthcare professional (35).

### 2.7 Statistical analysis

Per the guidelines established by NHANES (36), adjustments were made for unequal probabilities of selection, oversampling, and instances of nonresponse by utilizing stratification methods, sampling weights, and primary sampling units. The SII underwent a log transformation, subsequently classifying participants into quartiles based on their SII values. We analyzed categorical variables using counts and percentages, continuous variables with means (standard error, SE), and compared participants’ sociodemographic characteristics with ANOVA and Chi-square tests. A weighted logistic regression analysis was performed to assess the relationship between SII and the prevalence of CKD, during which the odds ratio and 95% confidence interval were computed. Moreover, an exploration of possible nonlinear associations was conducted utilizing restricted cubic spline analysis, with the p-values assessed through the Wald test. Survival curves corresponding to varying levels of SII and different causes of death were generated utilizing the svyjskm function from the jskm package, and weighted logrank tests were conducted to evaluate disparities in cumulative mortality rates. A Cox proportional hazards model with weighting was employed to evaluate the hazard ratio (HR) alongside the 95% confidence intervals (CIs) regarding the relationship between SII and the risk of death, while controlling for variables such as age, gender, ethnicity, marital status, education level, alcohol consumption, body mass index (BMI), income-to-poverty ratio, hypertension, diabetes, and various other adjustments (unadj.p, adj1.p, adj2.p). An analysis employing restricted cubic splines was performed to investigate possible non-linear connections between SII and the risk of mortality. To evaluate both overall and non-linear connections, the Wald test was employed to derive P-values. The assessments were performed across the entire cohort, encompassing both individuals with and without chronic kidney disease. An examination of the causes of mortality through RCS analysis revealed a significant threshold at SII. The logarithmic value is approximately equivalent to 6. This threshold was used to examine the relationship between SII.log and mortality risk on either side of the inflection point. To analyze the variability among different studies, the ‘metagen’ function from the ‘meta’ package in R was utilized to determine the p-value associated with heterogeneity. A significant interaction was identified, indicated by a p-value lower than 0.05. To mitigate the effects of potential reverse causality, analyses were conducted incorporating stratified adjustments as part of sensitivity evaluations. The following criteria were established to exclude certain cases from analysis: 1) individuals who passed away during the first 24 months of observation were excluded; 2) individuals younger than 45 years were excluded from the study. All statistical evaluations were performed utilizing R software, specifically version 4.3.3. A two-tailed P-value lower than 0.05 was deemed to be statistically significant unless stated otherwise.

### 2.8 Ethics declarations

NHANES offers a comprehensive overview of the health and nutritional conditions of individuals residing in non-institutional settings across the nation.S.civilians. Comprehensive details are available in the NHANES Methodology and Analysis Guide. The NHANES received approval from the Research Ethics Review Board of the National Center for Health Statistics. Informed consent was obtained from each individual participant.

## 3 Results

### 3.1 Characteristics of the participants

A total of 46,620 individuals were recruited for the study. As of December 31, 2019, the rate of mortality due to all causes stood at 10.54% (weighted percentage), which comprised 8,635 individuals who had received a diagnosis of chronic kidney disease. For those who unfortunately passed away, the rate of mortality among individuals with chronic kidney disease was recorded at 31.35% (weighted percentage). Participants were divided into four groups according to their baseline SII levels: Q1 (n=11,649), Q2 (n=11,663), Q3 (n=11,643), and Q4 (n=11,665). The initial attributes of the study group are detailed in **Table 1**. The largest share of participants consisted of individuals identified as Non-Hispanic White. The levels of existing and active smokers progressively rose alongside higher SII levels. Elevated levels of SII are linked to cardiovascular diseases and elevated blood pressure, though there is no correlation with diabetes. As SII levels rise, the share of patients suffering from CKD shows an upward trend, whereas the number of patients without CKD declines steadily. In general, patients were at G1, G2, and A1 levels, although G3a-G5 and A2-A3 levels correlated positively with SII levels. The majority of individuals demonstrating increased SII levels were primarily women, identified as Non-Hispanic White, married, with at least a high school diploma, belonging to the middle-income bracket, and showed patterns of alcohol use, along with elevated eGFR and diminished UACR metrics.

**Table 1:** Baseline characteristics according to SII levels on admission.Values are standard error (SE) or N (weighted percentage %)_a_.

Characteristics	Total (N=46620)	Q1 (N=11649)	Q2 (N=11663)	Q3 (N=11643)	Q4 (N=11665)	P value
Age	47.00 (0.18)	46.56 (0.26)	46.41 (0.22)	46.86 (0.24)	48.09 (0.26)	< 0.0001
SII	557.86 (2.87)	257.25 (0.73)	405.76 (0.45)	561.76 (0.63)	962.12 (4.69)	< 0.0001
Sex						< 0.0001
Female	23457 (51.05)	5317 (45.60)	5728 (49.19)	6028 (51.62)	6384 (56.99)	
Male	23163 (48.95)	6332 (54.40)	5935 (50.81)	5615 (48.38)	5281 (43.01)	
Race/ethnicity_b_						< 0.0001
Non-Hispanic White	20819 (68.83)	3860 (59.32)	5086 (68.96)	5646 (71.59)	6227 (74.14)	
Other Hispanic	3898 (5.62)	950 (5.43)	1043 (5.96)	994 (5.67)	911 (5.41)	
Non-Hispanic Black	9384 (10.60)	3829 (19.04)	2197 (9.69)	1764 (7.80)	1594 (7.02)	
other_c_	12519 (14.95)	3010 (16.21)	3337 (15.39)	3239 (14.93)	2933 (13.43)	
Poverty						< 0.001
<1.3	13988 (21.04)	3533 (21.63)	3398 (19.92)	3444 (20.26)	3613 (22.39)	
1.3 to <3.5	17092 (34.92)	4267 (35.10)	4220 (34.34)	4244 (34.76)	4361 (35.49)	
≥3.5	15540 (44.05)	3849 (43.27)	4045 (45.74)	3955 (44.98)	3691 (42.12)	
Education						< 0.0001
<High school	12536 (16.90)	3176 (17.84)	3126 (16.40)	3135 (16.57)	3099 (16.90)	
High school	10770 (23.92)	2605 (22.40)	2563 (22.51)	2743 (24.85)	2859 (25.70)	
>High school	23314 (59.18)	5868 (59.76)	5974 (61.09)	5765 (58.58)	5707 (57.40)	
Marital status						< 0.0001
Married or Living with partner	28283 (64.53)	7104 (65.51)	7325 (67.05)	7131 (64.72)	6723 (61.02)	
Divorced/Separated/Widowed/Never married_d_	18337 (35.47)	4545 (34.49)	4338 (32.95)	4512 (35.28)	4942 (38.98)	
BMI						< 0.0001
<25	13794 (31.13)	3681 (34.06)	3431 (31.67)	3266 (28.90)	3416 (30.28)	
25 to <30	15835 (33.45)	4097 (34.63)	4137 (35.36)	3930 (33.22)	3671 (30.80)	
≥30	16991 (35.42)	3871 (31.32)	4095 (32.97)	4447 (37.88)	4578 (38.92)	
Smoke						< 0.0001
no	25163 (53.66)	6578 (55.90)	6509 (55.58)	6262 (53.86)	5814 (49.64)	
yes	21457 (46.34)	5071 (44.10)	5154 (44.42)	5381 (46.14)	5851 (50.36)	
Alcohol.user						0.01
no	7525 (12.99)	1967 (13.99)	1950 (13.27)	1799 (12.34)	1809 (12.48)	
yes	39095 (87.01)	9682 (86.01)	9713 (86.73)	9844 (87.66)	9856 (87.52)	
DM						< 0.0001
no	38436 (87.23)	9671 (88.06)	9665 (87.98)	9689 (87.64)	9411 (85.38)	
yes	8184 (12.77)	1978 (11.94)	1998 (12.02)	1954 (12.36)	2254 (14.62)	
Hypertension						< 0.0001
no	26838 (63.27)	6860 (65.35)	6993 (66.09)	6725 (62.93)	6260 (59.06)	
yes	19782 (36.73)	4789 (34.65)	4670 (33.91)	4918 (37.07)	5405 (40.94)	
Cancer						< 0.0001
no	42324 (90.69)	10729 (91.47)	10690 (91.22)	10596 (91.23)	10309 (88.98)	
yes	4296 (9.31)	920 (8.53)	973 (8.78)	1047 (8.77)	1356 (11.02)	
CKD						< 0.0001
no	37985 (86.03)	9829 (88.13)	9756 (87.41)	9536 (86.65)	8864 (82.24)	
yes	8635 (13.97)	1820 (11.87)	1907 (12.59)	2107 (13.35)	2801 (17.76)	
CKD_prognosis						< 0.0001
low_risk	37985 (86.03)	9829 (88.13)	9756 (87.41)	9536 (86.65)	8864 (82.24)	
moderate_risk	5861 (10.17)	1278 (8.69)	1362 (9.78)	1460 (9.92)	1761 (12.07)	
high_risk	1712 (2.50)	356 (2.17)	342 (1.90)	396 (2.24)	618 (3.62)	
very_high_risk	1062 (1.30)	186 (1.01)	203 (0.91)	251 (1.18)	422 (2.07)	
CKD_ACR_e_						< 0.0001
A1	40685 (90.53)	10415 (92.07)	10362 (91.81)	10214 (90.98)	9694 (87.51)	
A2	4890 (8.06)	1047 (6.84)	1093 (7.25)	1192 (7.76)	1558 (10.22)	
A3	1045 (1.40)	187 (1.09)	208 (0.94)	237 (1.26)	413 (2.27)	
CKD_eGFR_f_						< 0.0001
G1	27172 (60.09)	6950 (61.67)	6974 (60.87)	6844 (60.36)	6404 (57.69)	
G2	15218 (33.44)	3817 (32.71)	3785 (33.26)	3753 (33.54)	3863 (34.16)	
G3a	2757 (4.49)	611 (4.14)	624 (4.38)	693 (4.24)	829 (5.16)	
G3b	1048 (1.46)	201 (1.16)	199 (1.09)	255 (1.36)	393 (2.16)	
G5	105 (0.12)	25 (0.09)	14 (0.08)	22 (0.09)	44 (0.21)	
G4	320 (0.40)	45 (0.23)	67 (0.32)	76 (0.40)	132 (0.62)	
Death_allcause						< 0.0001
0	39493 (89.46)	10203 (91.06)	10165 (91.39)	9935 (90.29)	9190 (85.37)	
1	7127 (10.54)	1446 (8.94)	1498 (8.61)	1708 (9.71)	2475 (14.63)	
Death_CVD_g_						< 0.0001
0	44367 (96.87)	11219 (97.54)	11181 (97.35)	11083 (97.10)	10884 (95.59)	
1	2253 (3.13)	430 (2.46)	482 (2.65)	560 (2.90)	781 (4.41)	
Death_cancer						< 0.0001
0	45032 (97.50)	11272 (97.56)	11316 (97.77)	11290 (97.80)	11154 (96.87)	
1	1588 (2.50)	377 (2.44)	347 (2.23)	353 (2.20)	511 (3.13)	

BMI, Body mass Index; DM, Diabetes mellitus; CKD, Chronic Kidney Disease; CVD, Cardiovascular disease; eGFR, Estimated Glomerular Filtration Rate; NHANES, National Health and Nutrition Examination Survey; PIR, Poverty Income Ratio; SE, Standard error; UACR, Urinary albumin-to-creatinine ratio.

a. All SEs for continuous variables and percentages for categorical variables were weighted.

b. Race and ethnicity were self-reported.

c. Includes multi-racial participants. NHANES does not provide a detailed list of all races and ethnicities.

d. Included widowed, divorced, separated individuals or never married.

e. UACR was divided into three levels: A1, <30mg/g or <3 mg/mmol; A2, 30-300 mg/g or 3-30 mg/mmol; A3, >300 mg/gor >30 mg/mmol.

f. eGFR was divided into five levels: G1, eGFR ≥90 ml/(min·1.73m2); G2, eGFR, 60-89 ml/(min·1.73m_2_); G3a, eGFR, 45-59 ml/(min·1.73m_2_); G3b, eGFR, 30-44 ml/(min·1.73m_2_); G4, eGFR, 15-29 ml/(min·1.73m_2_); G5, eGFR, <15ml/(min·1.73m_2_).

g. Includes congestive heart failure, coronary heart disease, angina, heart attack and stroke.

### 3.2 The relationship between SII and the occurrence of CKD

Logistic regression analyses concerning overall mortality are shown in **Supplementary Figure S2**. In the unadjusted model, the occurrence of CKD rose with an increase in SII (OR, 1.45, 95% CI: 1.36-1.56). For categorical variables, CKD prevalence was elevated in the Q3 group (OR 1.14, 95% CI: 1.04–1.26) and the Q4 group (OR 1.60, 95% CI: 1.45-1.77) relative to the Q1 group. The association persisted independently of potential confounders and remained statistically significant in Models 2 (adj1.p) and 3 (adj2.p) with p<0.001 (**Supplementary Figure S2**). It was particularly notable in the Q4 group, with odds ratios reported as 1.58 (95% CI: 1.43-1.76) and 1.51 (95% CI: 1.36-1.68), both with p<0.001. Our analysis using a smoothed spline curve modeling approach revealed a curvilinear association between SII.log and the progression of chronic kidney disease. The results showed a dose-response gradient, elevated levels of SII.log were associated with increased chances of developing chronic kidney disease (**Supplementary Figure S3**).

### 3.3 The relationships between SII and overall mortality, cardiovascular disease fatalities, and cancer-related deaths in patients with chronic kidney disease.

**Figure 1:**
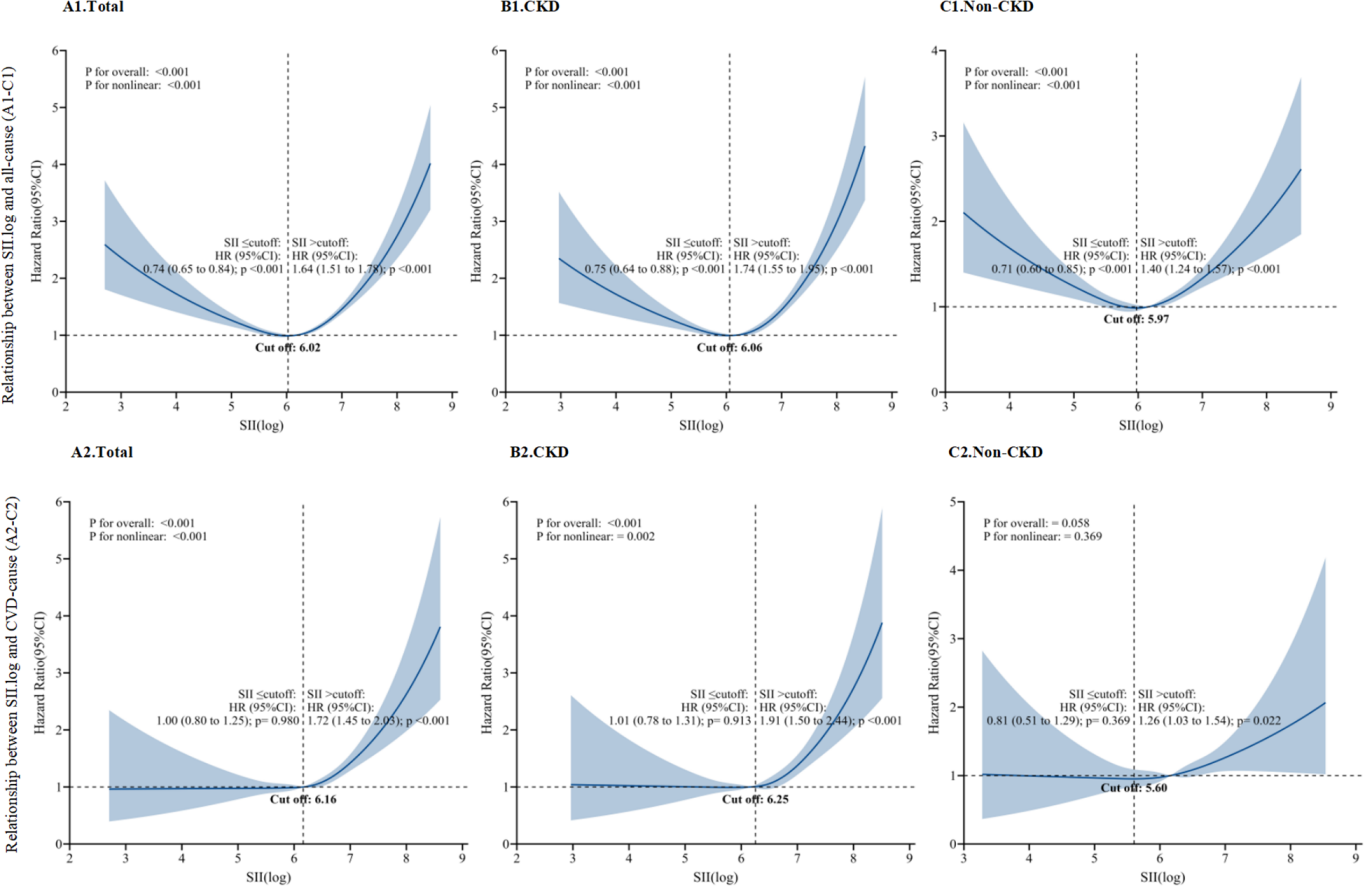
RCS fitting for the association between SII.log and all-cause **(A1-C1)** and CVD mortality **(A2-C2)**. (**A1**. Total; **B1**. CKD; **C1**. Non-CKD; **A2**. Total; **B2**. CKD; **C2**. Non-CKD).

In CKD patients, the total number of fatalities attributed to cardiovascular diseases reached 2,253; 1,588 cases of malignant tumors; and 7,127 all-cause deaths (**Supplementary Figure S4**). We present the weighted Cox regression results as forest plots after adjusting for various factors. The results showed that in Model 3, SII.log in the Q4 group was significantly associated with the all-cause mortality risk (HR, 1.25; 95% CI: 1.11 - 1.42) and CVD mortality risk (HR, 1.30; 95% CI: 1.06 - 1.59) in the CKD population. The association was independent of potential confounders, and the difference was statistically significant (p < 0.05). **Figure 1** demonstrates a strong non-linear correlation between SII.log and mortality rates from both all causes (HR 0.74; 95% CI: 0.65-0.84 and HR 1.64; 95%CI: 1.51–1.78) and cardiovascular diseases (HR 1.00; 95% CI: 0.80-1.25 and HR 1.72; 95%CI: 1.45–2.03) among patients(p<0.001) (**Figures 1A1, A2**). In CKD outpatients over 20, the SII.log demonstrated a U-shaped correlation with both overall and cardiovascular-related mortality rates, indicating that mortality risk can be heightened at both lower and higher SII levels(**Figures 1B1, B2**). In order to determine the significance of the inflection point values, a threshold effect analysis was performed, which indicated turning points at levels of 6.06 and 6.25, respectively(**Figures 1B1, B2**). The lowest mortality rates associated with all causes were observed at particular levels of SII.log. However, when SII.log exceeded these thresholds, the hazard for all-cause mortality increased significantly (HR 0.75; 95% CI: 0.64–0.88 and HR 1.74; 95% CI: 1.55–1.95), as well as for cardiovascular disease-related mortality (HR 1.01; 95% CI: 0.78–1.31 and HR 1.91; 95% CI: 1.50–2.44) (**Figures 1B1, B2**). SII.log value that surpasses 6.06 or 6.25 correlates with a 74% elevated risk of mortality due to all causes, and a 91% rise in death related to cardiovascular issues. However, SII.log did not show a significant impact on the cardiovascular mortality risk of individuals without chronic kidney disease, but it still maintained a correlation in all-cause mortality (**Figures 1C1, C2**). The cancer mortality rate in CKD patients also follows this pattern with SII.log (HR 0.64; 95%CI: 0.52–0.78 and HR 2.43; 95% CI: 1.51–3.93)(**Supplementary Figure S5B**).

**Figure 2:**
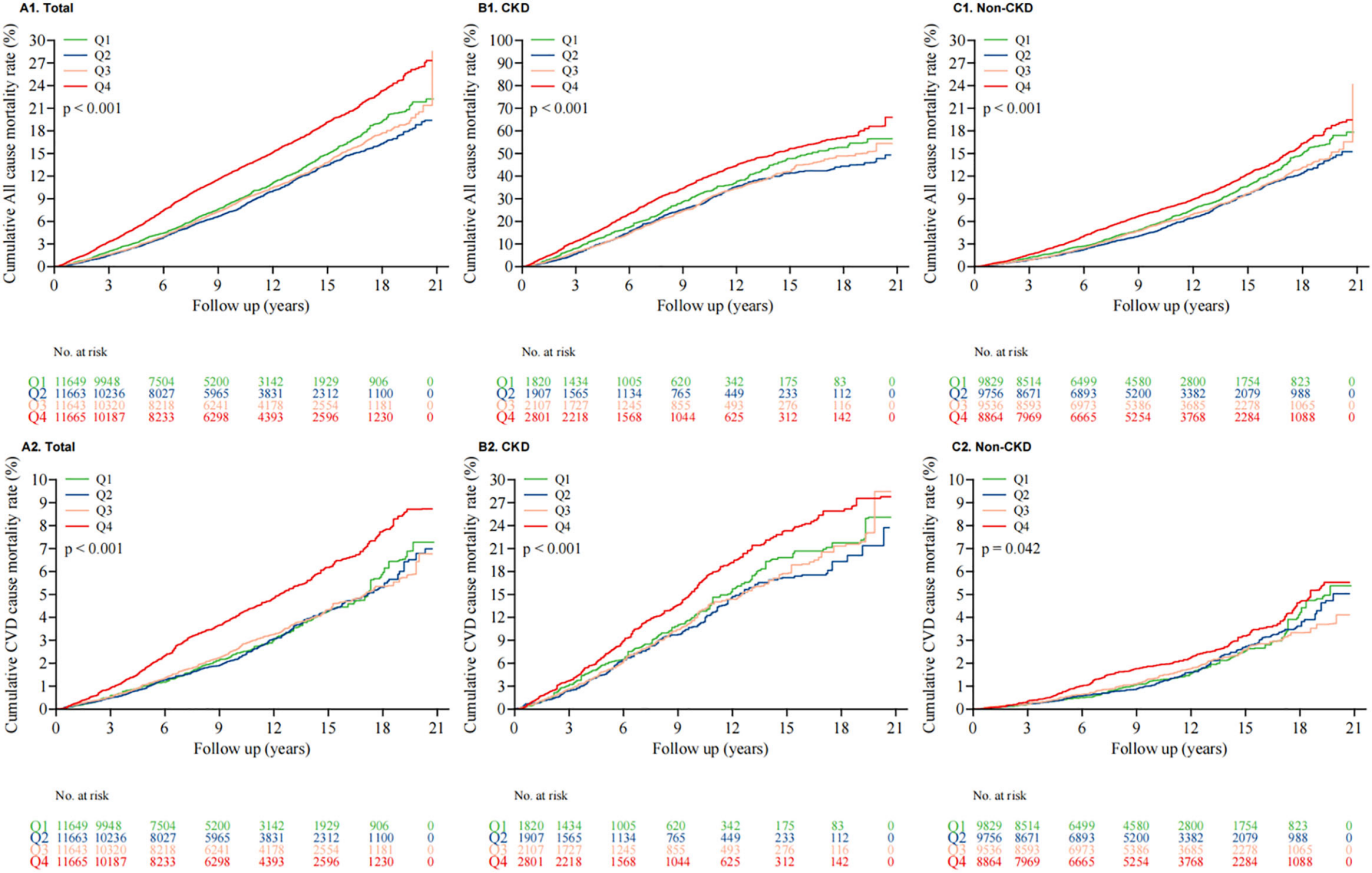
Kaplan-Meier survival curve for all-cause **(A1-C1)** and CVD mortality **(A2-C2)** of CKD individuals. **(A1**. Total; **B1**. CKD; **C1**. Non-CKD; **A2**. Total; **B2**. CKD; **C2**. Non-CKD).

According to the Kaplan-Meier survival analysis shown in **Figure 2**, in the risk of death due to cardiovascular diseases and overall causes, the Q4 quartile group shows that the survival period of CKD patients is shortened (**Figures 2A1, B1, A2, B2**), although there is no significant change in cancer-related deaths (**Supplementary Figure S6**). The results are statistically significant (p < 0.001), showing a notable SII.log dependence in group Q4. However, the relationship between SII.log and the cancer mortality rate remains significant in the overall cancer mortality (**Supplementary Figure S6A**). But when the population is divided into CKD and non-CKD groups, this correlation disappears (**Supplementary Figure S6B**). This is likely due to the smaller number of cancer deaths, which may have weakened this correlation. In the future, it is necessary to further expand the sample size to further explore the relationship between SII and cancer mortality in the CKD population.

**Figure 3:**
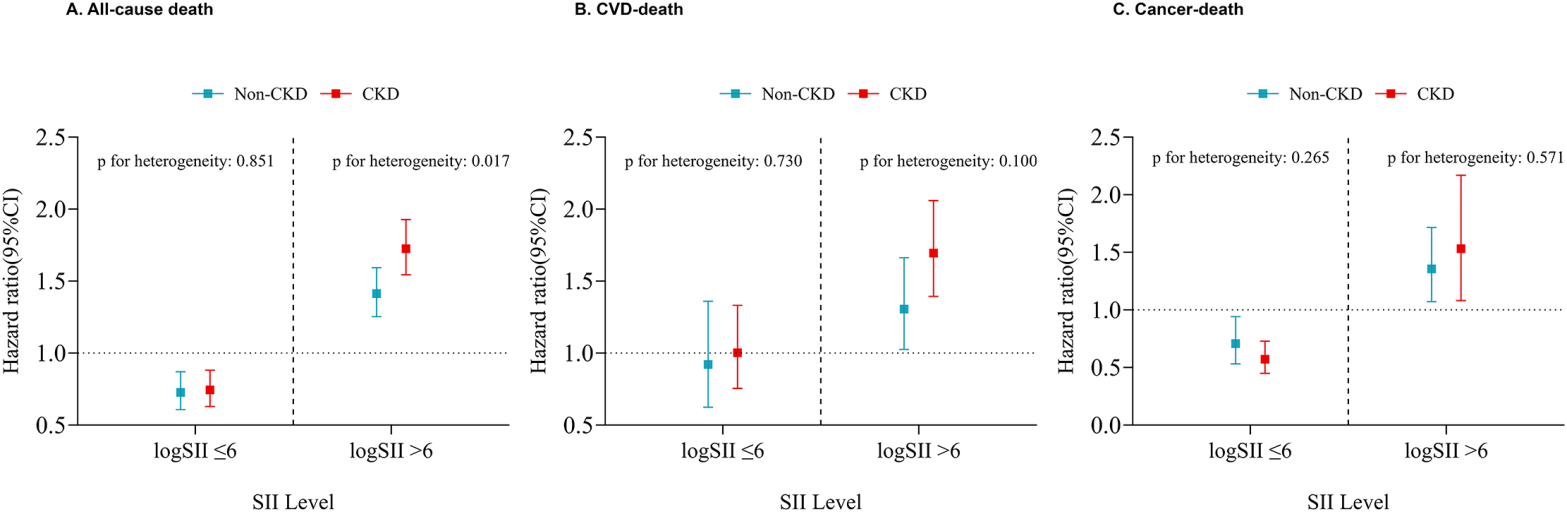
Stratified analysis was performed to further explore the association between SII.log and various causes of death. (**A**. All-cause death; **B**. CVD-death; **C**. Cancer-death).

Further exploration of the association between SII.log and various causes of death was conducted through stratified analysis (**Figure 3**). The population was stratified into CKD and non-CKD groups using a cutoff value of 6 for SII.log. This further clarified the impact of SII on various mortality scenarios in the CKD population. As shown in the figure, when SII.log > 6, the all-cause mortality rate in the CKD population significantly increased (P < 0.05) (**Figure 3A**). Although the same trend was maintained in CVD and Cancer deaths, there was no statistical difference (**Figures 3B, C**). Based on this result, we conducted a sensitivity analysis. As shown in **Supplementary Figure S7**, after excluding individuals who died within 2 years and those under 45 years old, SII.log was still associated with all-cause mortality in the CKD population (**Supplementary Figure S7A1, A2**). In the comparison of cardiovascular disease and cancer mortality rates in the CKD population, SII was more likely to increase the mortality rate of cardiovascular disease (**Supplementary Figures S7B1, B2, C1, C2**).

## 4 Discussion

The challenges posed by chronic kidney disease (CKD) patients create a considerable strain on healthcare providers and medical systems due to the complications and elevated risk of mortality associated with this condition (37). This innovative study sought to investigate the relationship between systemic inflammatory index (SII) and the death rates among individuals suffering from advanced chronic kidney disease (CKD), regardless of their dialysis status. The findings indicated that individuals over 45 had a more pronounced connection between SII and overall mortality. Additionally, the findings indicated a noteworthy correlation between SII and CKD specifically in female patients. This study explored the relationship between SII and various mortality rates in real-world hospitalized patients with CKD (23). This study suggests that tracking SII may help reduce general and specific cause mortality in patients with CKD (23). Clinically, SII is easy to obtain and convenient for long-term monitoring of CKD patients, and has been used for predicting mortality in various diseases, ischemic stroke and acute ST-segment elevation myocardial infarction (38, 39).A few investigations also have concentrated on the role of SII in forecasting mortality rates. The research conducted by Lai et al. revealed elevated levels of immune-related inflammation present at the time of admission were found to be an independent factor that raised the likelihood of mortality from all causes, cardiovascular issues, and cancer in hospitalized patients with chronic kidney disease (23). Previous research has indicated that the systemic immune-inflammation index (SII) serves as an indicator for both disease-free survival and overall longevity across several subtypes of breast carcinoma (40). Although the present study found that SII may be associated with an increased risk of cancer death, it was not significantly associated with the risk of cancer death in the CKD population. This is a difference from previous studies for reasons that, as previously mentioned, may be related to an insufficient sample of CKD patients dying. A recent study has shown that the global burden of diabetic kidney disease (DKD) has significantly increased over the past 32 years. For diabetic kidney disease caused by type 1 diabetes (DKD-T1DM), the number of new cases globally and the age-standardized years lived with disability (YLDS) have both increased significantly. For diabetic kidney disease caused by type 2 diabetes (DKD-T2DM), the number of new cases globally has increased by 167.2% from 1990 to 2021, and the ASIR has increased by 21.0% during the same period. These findings also indicate that the age of the population may be significantly associated with the onset of CKD (2).This study investigated the effect of systemic immune-inflammatory index on all-cause, cardiovascular, and cancer mortality in patients with chronic kidney disease, with a focus on those aged 45 years and older. The heightened risk of mortality among individuals with chronic kidney disease is closely associated with various pathophysiological factors such as persistent infections, inadequate nutrition, ongoing immunosuppressive therapies, accumulation of carcinogenic substances, and difficulties in DNA repair mechanisms (41–43). These elements amplify the inflammatory processes and hinder immune capabilities (44–46). The roles of neutrophils, platelets, and lymphocytes, both individually and collectively, highlight the potential impact of the systemic immune index on the survival rates of patients with chronic kidney disease. In cases of hypertension, neutrophils contribute to the impairment of endothelial function, leading to harm not only to the heart but also to the vascular system and renal tissues (47–49). Furthermore, the research conducted by Yvonne et al. indicates that neutrophils are responsible for initiating the formation of arterial wall plaques and also contribute to thrombosis. This has been proven to have a significant correlation with the mortality rates due to various causes and cardiovascular-related diseases in healthy men (50). Investigations show that platelets may protect circulating cancer cells from elimination and promote their metastasis by inducing a transition known as epithelial-mesenchymal change (51). The involvement of irregular activation of platelets and neutrophils has also been identified as a vital factor contributing to the occurrence of thrombosis (52, 53). At the same time, the decrease of lymphocytes may decrease the immune surveillance and immune defense function of the body, which may predispose to complications such as infection, further aggravate the disease and increase the risk of death. Lymphopenia has also been identified as a predictor of all-cause mortality in patients with acute kidney injury (AKI). Studies have shown that higher neutrophil-to-lymphocyte ratio (NLR) is associated with increased 30-day and 90-day mortality in patients with AKI. Lymphopenia and increased NLR together reflect immune dysregulation in AKI patients and are associated with an increased risk of death (54).These studies can assist in explaining the complexity of the relationship between SII and CKD prognosis.SII is a comprehensive index that encompasses neutrophils, platelets and lymphocytes. It is more comprehensive and accurate in predicting the risk of death from diseases.

Chronic inflammation is considered one of the important pathological mechanisms of cardiovascular diseases and many other diseases (55). As a new inflammatory marker, SII can reflect the body’s inflammatory state.In patients with CKD, the inflammatory response is usually more significant, which may be related to the accumulation of metabolic wastes due to decreased renal function, which can activate the immune system and trigger chronic inflammation (56). A study on psoriasis mentioned that psoriasis is a disease characterized by chronic inflammation. In this study, there was a statistically significant positive correlation between the SII/SIRI levels and the risk of psoriasis (57).This also suggests a possible association between SII and chronic inflammation.Finally, elevated SII may also interact with other risk factors in CKD patients, such as hypertension and diabetes, and increase cardiovascular burden through inflammatory pathways, further increasing the risk of all-cause mortality. This may explain the association between SII and all-cause and cardiovascular mortality in CKD patients found in our study. Therefore, SII, as a comprehensive inflammatory indicator, can provide important reference value for the prognosis evaluation of CKD patients (55, 56, 58).

The findings of this research also revealed a U-shaped relationship involving SII.log and prognosis in a CKD population aged > 20 years. As a comprehensive inflammatory indicator, SII.log makes the U-shaped connection analysis complex in the present study. The presence of high blood pressure and clot formation is well acknowledged as significant factors contributing to mortality among individuals with cardiovascular diseases. Further research by Giuseppe et al. has indicated that heightened levels of platelets are associated with an increased likelihood of mortality and cardiovascular incidents, suggesting that platelets might serve as a non-invasive indicator for assessing the risk of CVD, especially in terms of mortality (59). The diverse functions of platelets contribute to their varying roles across different diseases. In the context of an inflammatory response, low platelet and lymphocyte counts are associated with an increased risk of bleeding and impaired immune function. The role of lymphocytes in suppressing tumors involves triggering death in cytotoxic cells while simultaneously restraining the proliferation and movement of tumor cells (60). Platelets facilitate cancer cell proliferation by promoting tumor progression and metastasis (61, 62). Conversely, lymphocytes are crucial in the immune system for inhibiting tumor development by eliminating cancer cells and preventing their dissemination (63). Drawing on numerous prior studies, the systemic immune-inflammatory index (SII) may be directly associated with the prognosis of various diseases (18, 23, 64–66) including coronary artery disease, acute ischemic stroke, and malignancies.

Among patients with chronic kidney disease (especially those undergoing peritoneal dialysis), those with higher SII levels have a significantly increased risk of death (25). The reduction of SII may indicate the dysfunction of immune function. Studies have shown that in patients with chronic kidney disease, the active FGF23 signaling pathway may lead to impaired recruitment of neutrophils, thereby weakening the host defense ability (67). In addition, SII is also associated with short-term mortality risk in patients with acute kidney injury (AKI).At the same time, in the study of sepsis-associated acute kidney injury, a high or low SII value was associated with an increased risk of death within 28 days (68).This further suggests that changes in SII may be closely related to changes in prognosis in CKD patients. The above complex immunological mechanisms may provide a corresponding explanation for the U-shaped relationship. Taken together, both excessive and inadequate immune responses may cause damage to overall health. Therefore, an improved understanding of the mortality risk faced by patients with CKD could facilitate the development of tailored treatment strategies in the clinical setting. Integrating SII with established risk determinants appears to improve clinical prediction accuracy for long-term outcomes. Ultimately, more extensive future studies are needed to validate the predictive effect of SII on mortality in patients with CKD and to explore in depth the pathways through which SII affects mortality in patients with CKD.

### 4.1 Strengths and weaknesses

This study first identifies the relationship between SII and mortality rates among individuals over the age of 45 suffering from chronic kidney ailments. The association between the Systemic Immune-Inflammation Index (SII) and all-cause mortality in chronic kidney disease (CKD) patients remained significant, even after excluding those under 45 years old. The NHANES survey adhered to stringent protocols in its methodology, data gathering, and analytical processes, effectively reducing errors related to both sampling and measurement. An extensive evaluation was conducted on various mortality factors, utilizing regression models and stratified methods to explore additional elements influencing each specific cause. Nonetheless, there are certain constraints present in the research. To begin with, there was insufficient longitudinal observation of baseline SII values, and any variations during the follow-up phase were not documented. Secondly, factors such as tobacco use, alcohol intake, and socioeconomic status were provided by participants themselves, which may have introduced unchecked memory biases. Although we adjusted for some potential confounding factors through statistical methods, we cannot completely rule out the possibility that residual and uncontrolled confounding factors might explain the correlation. This is the limitation of this study. The eGFR was assessed using the CKD-EPI formula rather than the inulin-based reference method, and the UACR was utilized instead of measuring protein through 24-hour urine collection, which may lead to misclassification of CKD patients. This study was conducted based on an open database. The issue of insufficient sample size may affect the correlation between SII and cancer, as well as cardiovascular mortality. Therefore, in the future, more real-world studies are needed to further explore the relationship between SII and all-cause mortality, cardiovascular mortality, and cancer mortality in the CKD population.

## 5 Conclusion

For adults aged 45 and older who have chronic kidney disease, the systemic inflammation index is associated with an increased risk of all cause death. These results improve the ability to forecast outcomes based on systemic immune-inflammation measurements in chronic kidney disease patients while also introducing fresh insights into treatment approaches for this group.

## Data Availability

The original contributions presented in the study are included in the article/**Supplementary Material**. Further inquiries can be directed to the corresponding authors.

## Supplementary material

Supplementary Figure 1 Flow diagram of the selection of eligible participants.

Supplementary Figure 2 Association between SII.log and CKD.

Supplementary Figure 3 RCS fitting for the association between SII.log and CKD prevalence.

Supplementary Figure 4 Dose-response relationship between SII.log and various causes of death.

Supplementary Figure 5 RCS fitting for the association between SII.log and Cancer mortality **(A-C)**. (**A**. Total; **B**. CKD; **C**. Non-CKD).

Supplementary Figure 6 Kaplan-Meier survival curve for Cancer mortality of CKD individuals. (**A**. Total; **B**. CKD; **C**. Non-CKD).

Supplementary Figure 7 Stratified analysis was performed to explore the association between SII and various causes of death. (**A1**. All-cause death; **B1**. CVD-death; **C1**. Cancer-death; **A2**. All-cause death; **B2**. CVD-death; **C2**. Cancer-death).
