# Supplementary figures and images for: Systemic immune inflammatory index and mortality in chronic kidney disease
Source: Front Endocrinol (Lausanne). 2025 Sep 3;16:1605543. doi: 10.3389/fendo.2025.1605543 (PMC12442490; doi:10.3389/fendo.2025.1605543)

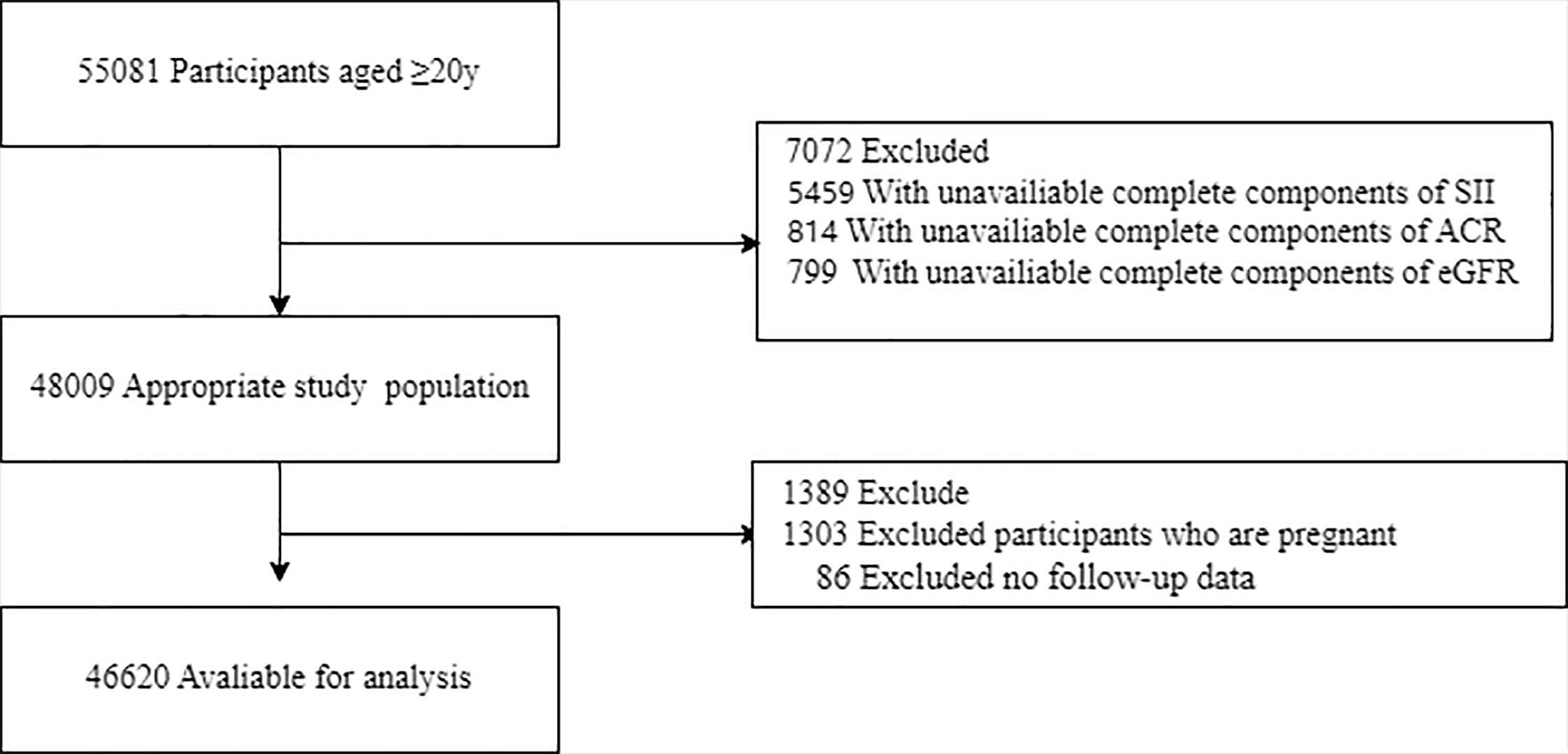

Supplement: Supplementary Figure 1 — Flow diagram of the selection of eligible participants. [file Image1.tiff]

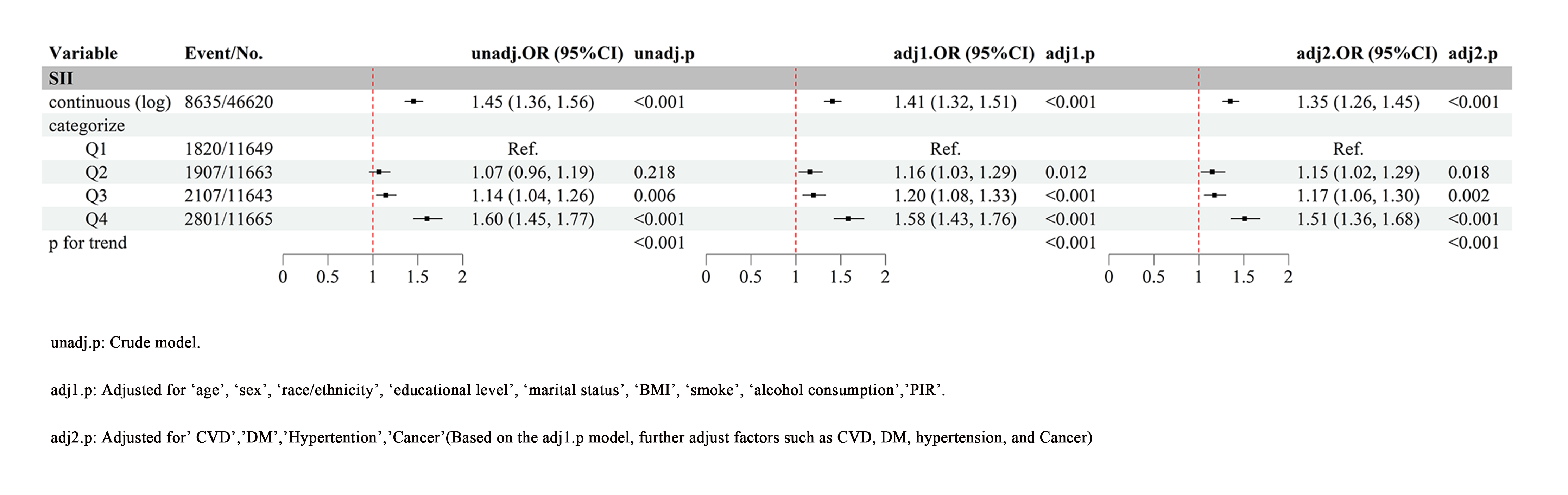

Supplement: Supplementary Figure 2 — Association between SII.log and CKD. [file Image2.tif]

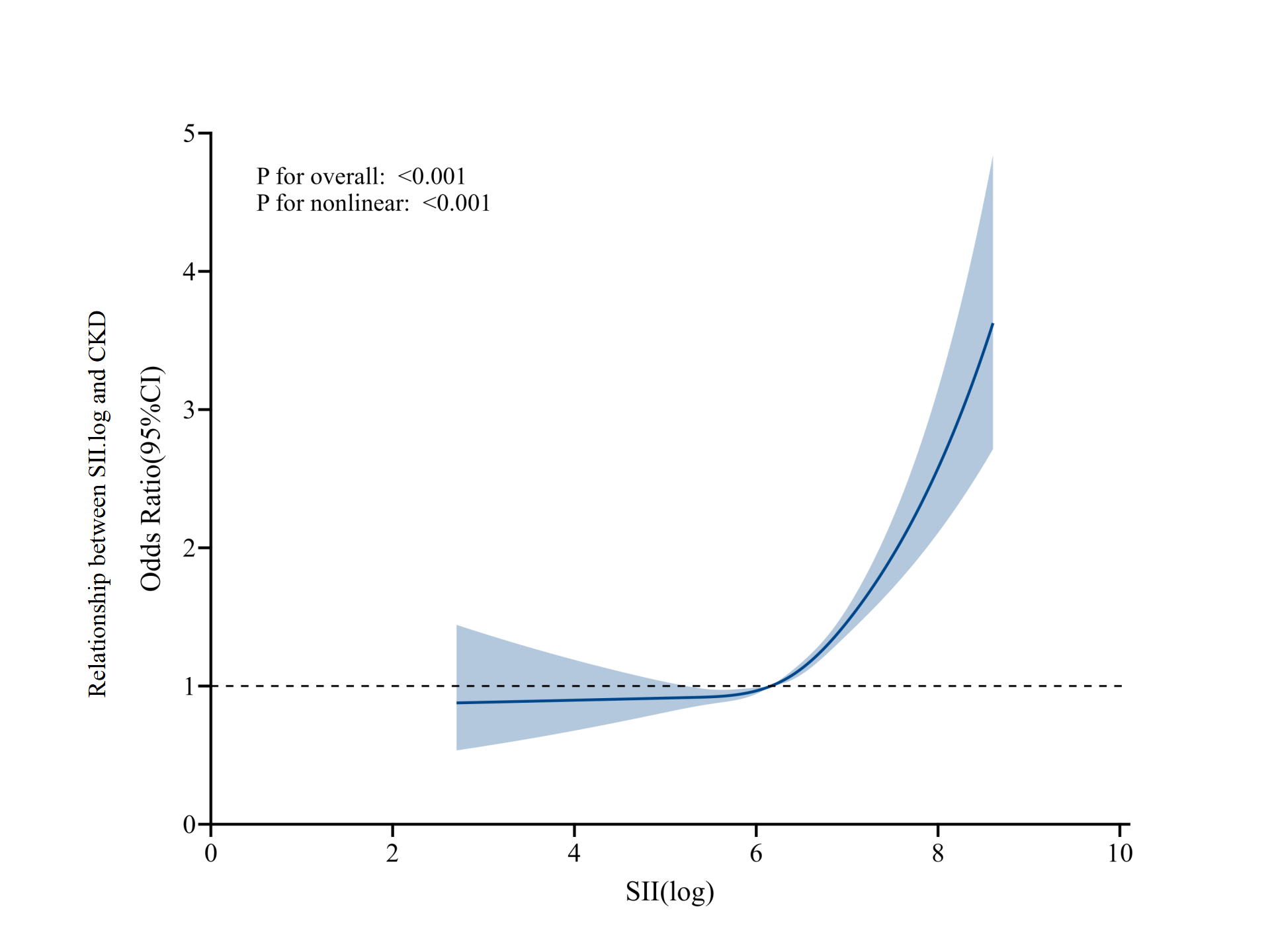

Supplement: Supplementary Figure 3 — RCS fitting for the association between SII.log and CKD prevalence. [file Image3.tif]

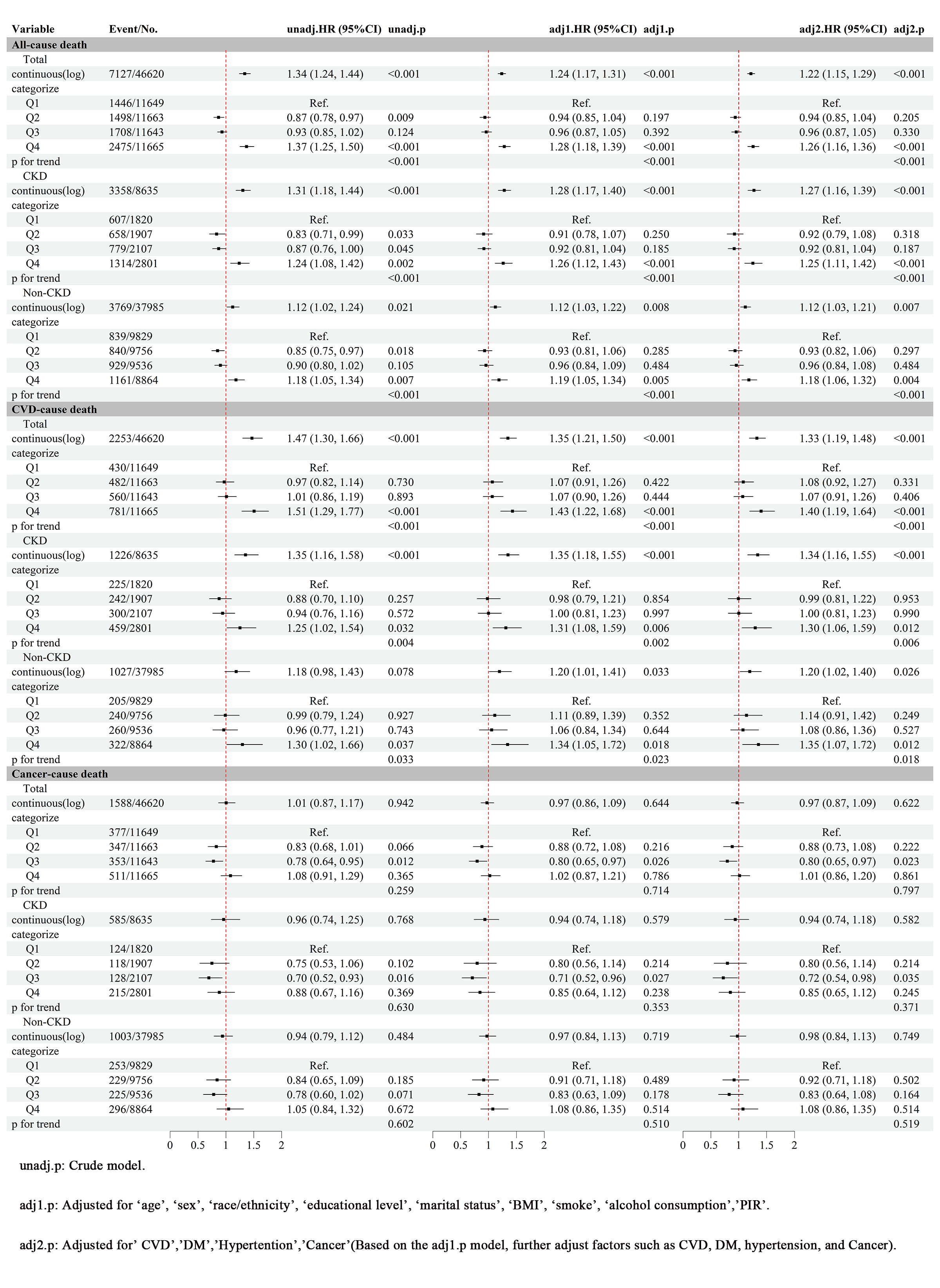

Supplement: Supplementary Figure 4 — Dose-response relationship between SII.log and various causes of death. [file Image4.tif]

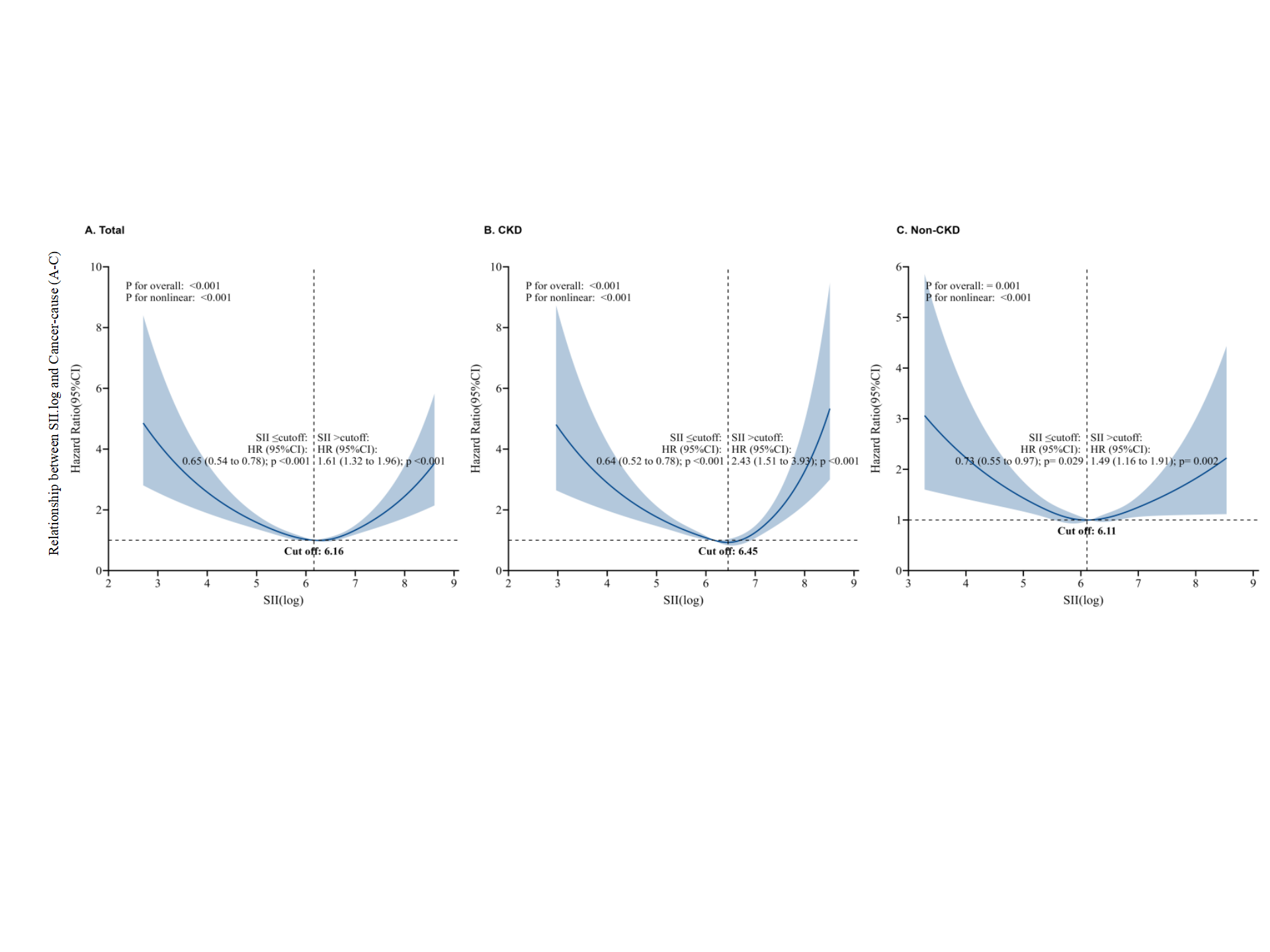

Supplement: Supplementary Figure 5 — RCS fitting for the association between SII.log and Cancer mortality (A-C). (A. Total; B. CKD; C. Non-CKD). [file Image5.tif]

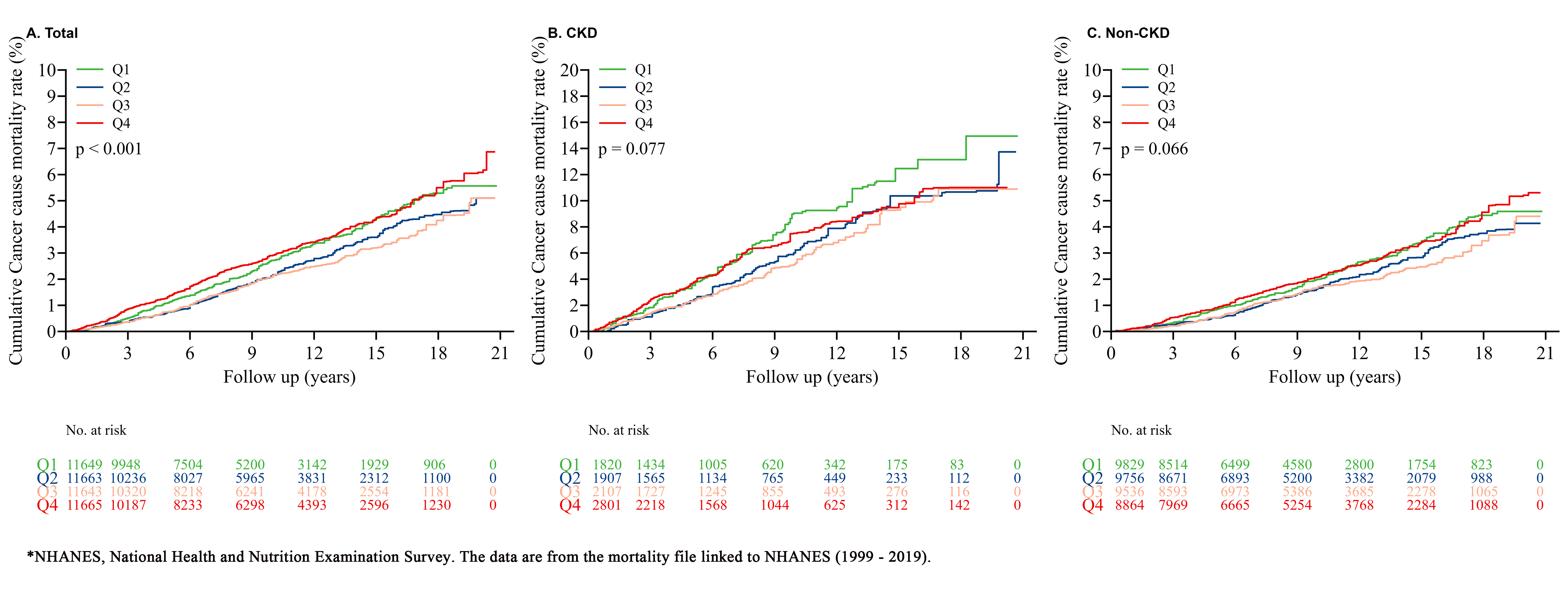

Supplement: Supplementary Figure 6 — Kaplan-Meier survival curve for Cancer mortality of CKD individuals. (A. Total; B. CKD; C. Non-CKD). [file Image6.tif]

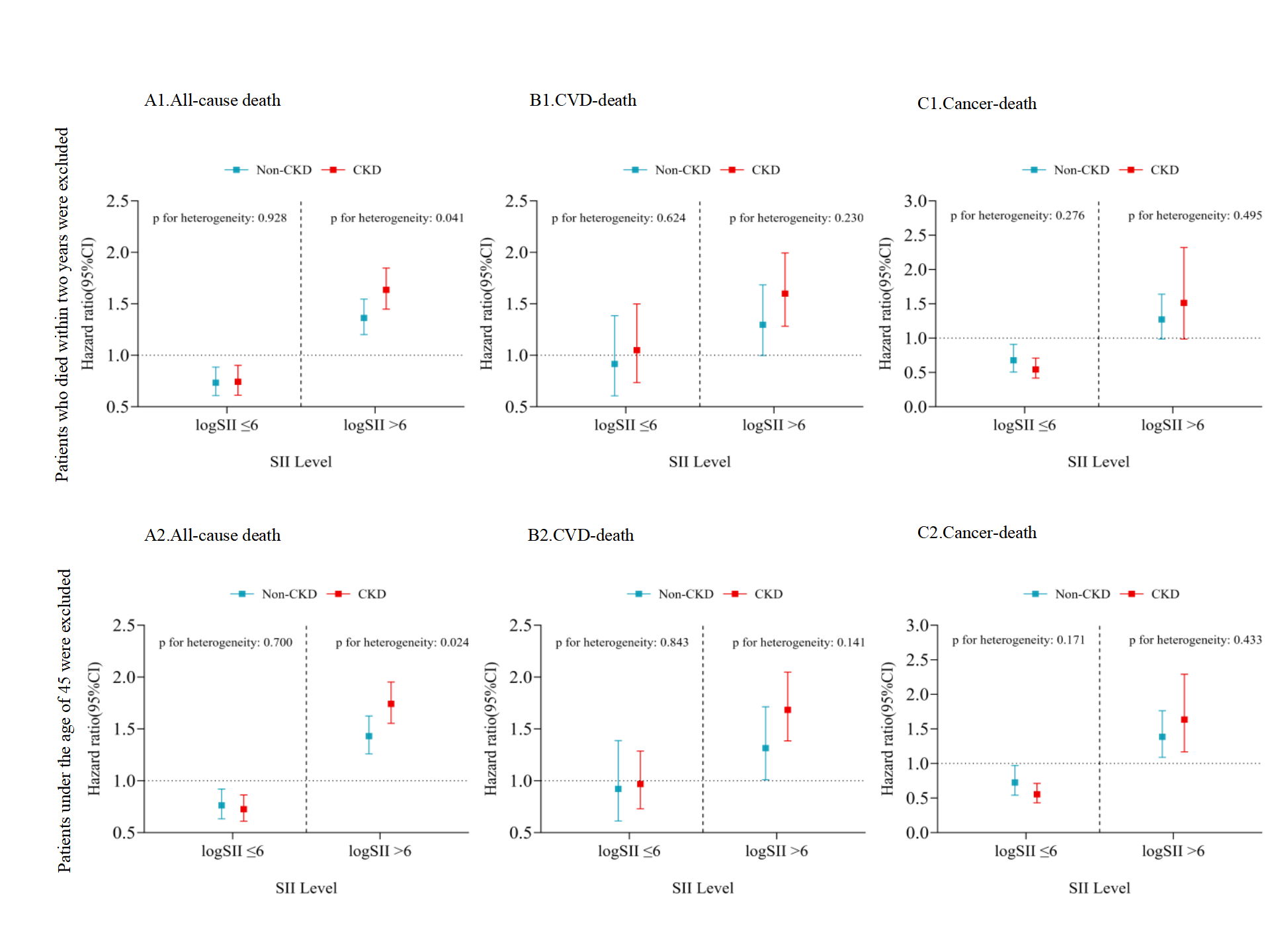

Supplement: Supplementary Figure 7 — Stratified analysis was performed to explore the association between SII and various causes of death. (A1. All-cause death; B1. CVD-death; C1. Cancer-death; A2. All-cause death; B2. CVD-death; C2. Cancer-death). [file Image7.tif]
